# Editorial: Resolution and regeneration of inflammation in lung and brain disorders

**DOI:** 10.3389/fimmu.2023.1291087

**Published:** 2023-10-03

**Authors:** Guojun Qian, Nu Zhang, Hongwei Fang, Hao Fang

**Affiliations:** ^1^ Department of Anesthesiology, Minhang Hospital, Fudan University, Shanghai, China; ^2^ Affiliated Cancer Hospital and Institute of Guangzhou Medical University, Guangzhou, China; ^3^ Department of Anesthesiology, Zhongshan Hospital, Fudan University, Shanghai, China; ^4^ Fudan Zhangjiang Institute, Shanghai, China

**Keywords:** inflammation, inflammation resolution, regeneration, lung disorders, brain, disorders

Our understanding of inflammation continues to evolve, revealing its intricate double-edged nature in human health. Once seen as a simple reaction, inflammation is now recognized as a complex multi-phase process aimed at restoring tissue homeostasis (1, 2). A key breakthrough is recognizing that the resolution phase, which ends inflammation, is an active and highly regulated process rather than a passive winding down (3). This special Research Topic, “*Resolution and Regeneration of Inflammation in Lung and Brain Disorders*”, navigates the winding path of inflammation from start to finish, highlighting its divergent outcomes across various lung and brain disorders.

The journey begins in the eye, where piperlongumine emerges as a promising agent against corneal transplant rejection. Beyond known therapeutic uses (4), Fan et al. elucidate that piperlongumine adeptly attenuates pathological angiogenesis and inflammation, thereby alleviating corneal allograft rejection. Despite its nascent preclinical status, its potential is worth investigating in clinical studies.

Moving to the lungs, idiopathic pulmonary fibrosis (IPF) manifests as a convoluted interplay between epithelium/fibroblast and the overarching immune milieu (5). Lin et al. identified aberrant gene signatures linked to abnormal neutrophil activity as a hallmark of IPF. Defects in both innate and adaptive immunity likely contribute to IPF pathogenesis, underscoring these genes as potential diagnostic markers and immunomodulatory treatment targets.

In acute respiratory distress syndrome (ARDS), neutrophil extracellular traps (NETs) reveal a sinister side, exacerbating lung injury by triggering macrophage pyroptosis (6). Liu et al. propose alpha-linolenic acid as a shield against NETs by modulating pyrin inflammasome activity, unveiling promising therapeutic strategies against sepsis-induced ARDS.

Even life-saving interventions like mechanical ventilation can have adverse effects, potentially initiating lung fibrosis (7). Tang et al. implicate non-coding RNA abnormalities in ventilation-induced injury, spotlighting the intricate molecular underpinnings of this iatrogenic complication.

Premature neonates also suffer the consequences of unchecked inflammation, often developing bronchopulmonary dysplasia (BPD) (8). Cui et al. demonstrate that prolonged exposure to lipopolysaccharide (LPS) in neonatal mice activates IL-17a^+^ lymphocytes, resulting in heightened pulmonary inflammation and alveolar damage. Remarkably, strategic interventions targeting the IL-17A axis and NKG2D pathways considerably mitigate LPS-induced pulmonary damages, underscoring their importance in the pathogenesis of BPD. These findings, obtained in neonatal investigations, may indeed provide insights for new therapeutic strategies.

In summary, this Research Topic describes new research paradigms, clarifying the inflammatory network in these diseases.

## Author contributions

GQ: Writing – original draft, Writing – review & editing. NZ: Writing – original draft. HWF: Writing – original draft. HF: Writing – review & editing.
